# Pipeline of Known Chemical Classes of Antibiotics 

**DOI:** 10.3390/antibiotics2040500

**Published:** 2013-12-06

**Authors:** Cristina d’Urso de Souza Mendes, Adelaide Maria de Souza Antunes

**Affiliations:** 1 Graduate Program in Technology of Chemical and Biochemical Processes, Technology Center, Federal University of Rio de Janeiro (UFRJ), EQ/UFRJ, Centro de Tecnologia, Bloco E, Ilha do Fundão, Rio de Janeiro-RJ 21949-900, Brazil; E-Mail: aantunes@inpi.gov.br; 2 Brazilian National Institute of Industrial Property, INPI/Rua Mayrink Veiga No. 9/19 andar, CEP 20090-910, Rio de Janeiro-RJ 20090-910, Brazil

**Keywords:** antibacterial, antibiotic, clinical trials, R&D

## Abstract

Many approaches are used to discover new antibiotic compounds, one of the most widespread being the chemical modification of known antibiotics. This type of discovery has been so important in the development of new antibiotics that most antibiotics used today belong to the same chemical classes as antibiotics discovered in the 1950s and 1960s. Even though the discovery of new classes of antibiotics is urgently needed, the chemical modification of antibiotics in known classes is still widely used to discover new antibiotics, resulting in a great number of compounds in the discovery and clinical pipeline that belong to existing classes. In this scenario, the present article presents an overview of the R&D pipeline of new antibiotics in known classes of antibiotics, from discovery to clinical trial, in order to map out the technological trends in this type of antibiotic R&D, aiming to identify the chemical classes attracting most interest, their spectrum of activity, and the new subclasses under development. The result of the study shows that the new antibiotics in the pipeline belong to the following chemical classes: quinolones, aminoglycosides, macrolides, oxazolidinones, tetracyclines, pleuromutilins, beta-lactams, lipoglycopeptides, polymyxins and cyclic lipopeptides.

## 1. Introduction

The continuous rise in the number of bacterial pathogens with resistance to the antibiotics on the market has prompted the urgent need for the development of new antibiotics. Currently there are three different approaches used in the discovery of new antibiotics: (1) development of new compounds that belong to new chemical classes and act on new targets; (2) development of new compounds that belong to new chemical classes and act on established targets; and (3) development of compounds belonging to existing classes that act on established targets [1].

However, the discovery of new chemical classes is a risky process [2]. Therefore, many companies developing new antibiotics are still pursuing the chemical modification of known antibiotics.

In this scenario, the present article presents an overview of the R&D pipeline of new antibiotics that belong to known classes of antibiotics, from discovery to clinical trials. With the information gathered, the article maps out the technological trends in this type of antibiotic R&D, identifying the chemical classes in which there is most R&D activity, the spectra of activity, the new subclasses, and the companies researching these types of compounds.

This article presents the different stages of R&D of companies developing new compounds in known chemical classes. Several articles [3,4,5,6,7,8,9] review the new antibiotic compounds in the clinical pipeline by analyzing their activity, technical and pharmacological properties. The contribution this article makes is to provide more wide-ranging information on the R&D pipeline at every phase of development, especially at pre-clinical and discovery stages, when there is little technical information available.

It is important to note that substances from the same chemical group have similar mechanisms of action and normally have the same basic structure, or scaffold, which is what lends the compounds their activity. The differences between the substances from the same group are caused by the peripheral chemical groups, which improve their pharmacological properties and can improve potency and antibacterial spectrum.

## 2. Experimental

The study consists of two stages, namely:

1. Identification of companies doing research and development into new antibiotics around the world;
2. Mapping of the antibiotic development pipelines of the companies identified.

The two stages will be developed using the following methodology:

### 2.1. Identification of Companies Doing R&D into New Antibiotics

The process of developing a new drug normally involves two major stages: the initial discovery stage and pre-clinical testing (*in vitro* and *in vivo* tests to determine efficacy, pharmacology, safety, *etc.*) and the clinical trial stage (involving trials on humans) [10,11].

Very little of the information produced at the discovery stage is published. When companies begin the clinical trials, they have to register the information in clinical trials register databases like clinicaltrials.gov, EU Clinical Trials Register (EU-CTR) and others, following the rules established by the World Health Organization [10,11].

As so little information is available on the discovery stage, this study identifies the companies working in the research and development of new antibiotics based on press releases and presentations by these companies in the business and health media. This information on the pharmaceuticals market is available on a global level and consolidated in the Chemical Business NewsBase (CBNB) database, which was chosen to be used in this study [12].

This database was mined for references with the words “antibacterial” or “antibiotic” in the R&D category published between 2006 and 2012. By analyzing these articles, companies working in the research and development of new antibiotics were identified.

The EvaluatePharma^®^ database was used for each of the companies to see whether any of them had gone bankrupt, gone out of business or been acquired by other companies in the period under study. 

### 2.2. Mapping the Companies’ New Antibiotics Pipeline

In this stage, the antibiotics developed by the companies and their stage of development (discovery phase, clinical trials, *etc.*) were identified. These data were collected from the companies’ own websites, from news published on the internet, articles retrieved from CBNB, scientific journals, patent applications and the clinical trial databases clinicaltrial.gov, ClinicalTrials.gov, a registry and results database of clinical studies of human participants conducted around the world, and International Clinical Trials Registry Platform (run by the WHO), that provides access to a central database containing the trial registration data sets provided by more than 15 registry databases. It also provides links to the full original records.

## 3. Pipeline of New Antibiotics in Chemical Classes Known on the Market

The chemical classes of antibiotics identified in the present study were: quinolones, aminoglycosides, macrolides, oxazolidinones, tetracyclines, pleuromutilins, beta-lactams, lipoglycopeptides, polymyxins, and cyclic lipopeptides. All the compounds, their phase of development, chemical class, and spectrum of activity are disclosed in Table 1.

**Table 1:** Pipeline of antibiotics in known chemical classes.

Class	Compound (Subclass)	Company (Country)	Development Phase	Bacteria	Chemical structure	Source
**Quinolones**						
	Nemonoxacin	TaiGen Biotechnology (Taiwan)	Phase III	Broad-spectrum	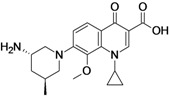	[13,14,15,16,17,18]
	Ozenoxacin (topical use)	Ferrer Internacional (Spain)	Phase III	Gram-positive	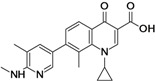	[19,20,21]
	Zabofloxacin (**fluoroquinolone**)	Dong Wha Pharmaceutical (South Korea)	Phase III	Broad-spectrum	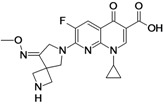	[22,23,24,25]
	Delafloxacin (**fluoroquinolone**)	Rib-X Pharmaceuticals (USA)	Phase III	Broad-spectrum	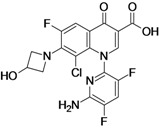	[26,27,28]
	Avarofloxacin (**fluoroquinolone**)	Furiex Pharmaceuticals (USA)	Phase II completed	Broad-spectrum	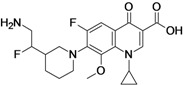	[29,30,31,32,33,34,35]
	Finafloxacin (**fluoroquinolone**)	MerLion Pharmaceuticals (Singapore)	Phase II	*H. pylori*, Broad-Spectrum	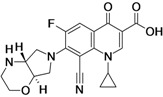	[36,37,38,39]
	WCK 2349 a prodrug of WCK 771 ^a,b^ (**fluoroquinolone**)	Wockhardt ( India)	Phase II	Gram-positive	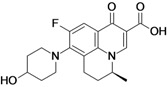 *S*-(−)-nadifloxacin ^b^	[40,41,42,43]
	KRP-AM1977X (oral)	Kyorin (Japan)	Phase I	Respiratory infections	ND	[44,45]
	KRP-AM1977Y	Kyorin	Phase I	MRSA	ND	[44,45]
	DS-8587 (**fluoroquinolone**)	Daiichi Sankyo (Japan)	Phase I	Broad-spectrum	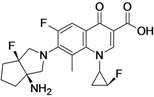	[46,47]
	ACH-702 (**Isothiazoloquinolone**)	Achillion Pharmaceuticals (USA)	Pre-Clinical	Broad-spectrum	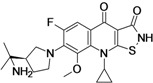	[48,49]
**Aminoglycosides**						
	Plazomicin	Achaogen (USA)	Phase II completed	Broad-spectrum	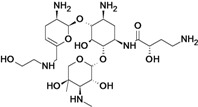	[50,51,52,53,54,55,56]
	Neomycin analogs ^c^	Achaogen (USA)	Discovery	Broad-spectrum	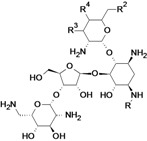	[57,58,59,60,61,62,63]
	FY-901	Changzhou Fangyuan Pharmaceutical (China)	Pre-Clinical	*S. Aureus*	ND	[64]
	FY-902	Changzhou Fangyuan Pharmaceutical	Pre-Clinical	*M. tuberculosis*	ND	[64]
	New compounds	Changzhou Fangyuan Pharmaceutical	Discovery	ND	ND	[65]
**Macrolides**						
	Solithromycin (2-fluoroketolide)	Cempra Pharmaceuticals (USA)	Phase III	*S. pneumoniae; N. gonorrhoeae*	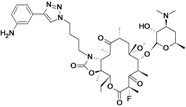	[66,67,68,69,70,71,72]
	New Macrolides ^d^	Cempra Pharmaceuticals	Discovery	ND	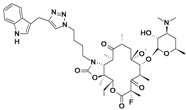	[73,74]
	EDP-788 (bicyclolide-bridged bicyclic macrolide)	Enanta Pharmaceuticals (USA)	Pre-Clinical	Gram-positive	ND	[75,76]
	new bicyclolides ^c^	Enanta Pharmaceuticals	Discovery	Gram-positive	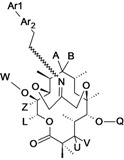	[77,78,79,80]
	WCK 4873 (Ketolide)	Wockhardt	Pre-Clinical	MDR *pneumoccocus*, *Strep*, *H. influenzae*	ND	[81,82]
	RX-02 program	Rib-X Pharmaceuticals	Discovery	Gram-positive	ND	[83]
**Oxazolidinones**						
	Tedizolid	Cubist Pharmaceuticals (USA)	Phase III	Gram-positive	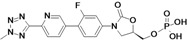	[84,85,86,87,88]
	Cadazolid	Actelion (Switzerland)	Phase II completed	*Clostridium difficile*	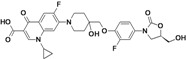	[89,90,91,92]
	Radezolid	Rib-X Pharmaceuticals	Phase II completed	Gram-positive	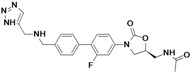	[93,94,95,96,97,98]
	AZD5847	AstraZeneca (UK)	Phase II	*M. tuberculosis*	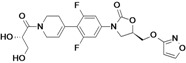	[99,100,101,102]
	LCB01-0371	LegoChem Biosciences (South Korea)	Phase I	Gram-positive	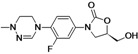	[103,104]
	MRX-I	MicuRx Pharmaceuticals (USA)	Phase I	Gram-positive	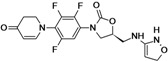	[105,106]
	MRX-II	MicuRx Pharmaceuticals	Pre-Clinical	Gram-positive	ND	[106]
	2nd Generation Oxazolidinones	Wockhardt	Pre-Clinical	Gram-positive	ND	[81]
**Tetracyclines**						
	Omadacycline (aminomethylcycline)	Paratek (USA)	Phase III	Broad-spectrum	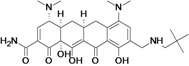	[93,107,108]
	Aminomethylcyclines	Paratek	Discovery	*Clostridium difficile*	ND	[107]
	Eravacycline (fluorocycline)	Tetraphase Pharmaceuticals (USA)	Phase II (cIAI)	Gram-negative	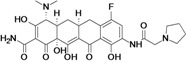	[109,110,111,112]
	TP-271	Tetraphase Pharmaceuticals	Pre-Clinical	bacterial biothreats, CABP pathogens	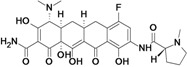	[109,113,114]
	TP-834	Tetraphase Pharmaceuticals	Pre-Clinical	CABP pathogens	ND	[109]
	New tetracycline derivatives ^c^	Tetraphase Pharmaceuticals	Discovery	Gram-negative	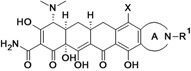	[115,116,117,118]
**Pleuromutilins**						
	BC-3781	Nabriva (Austria)	Phase II Completed	Gram-positive, including MRSA	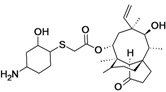	[119,120,121,122,123,124]
	BC-7013 (topical)	Nabriva	Phase I	Gram-positive	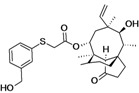	[119,124]
	Discovery/development of pleuromutilins	Nabriva	Discovery	Broad spectrum	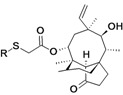	[119,125,126,127]
**Beta-lactams**						
	Ceftolozane + tazobactam (CXA-201)	Cubist Pharmaceuticals	Phase III	Gram-negative	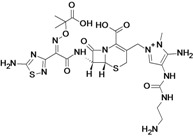 (ceftolozane)	[6,128,129,130,131,132]
	BAL30072 (sulfactam)	Basilea Pharmaceutica (Switzerland)	Phase I	Gram-negative	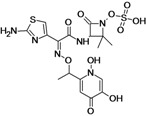	[133,134,135]
	S-649266 (GSK2696266) (Cephalosporin)	Shionogi (Japan)/ GlaxoSmithKline (UK)	Phase I	Gram-negative	ND	[136,137,138]
	CB-027 (Cephalosporin)	Cubist Pharmaceuticals	Pre-Clinical	Broad-spectrum	ND	[139]
	FSI-1671 (Carbapenem)	FOB Synthesis, Inc. (USA)	Pre-Clinical	Gram-negative	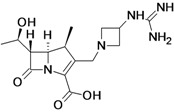	[140,141]
**Beta-lactamase inhibitors—Diazabicyclooctanes (DBOs)**						
	Ceftazidime/Avibactam (CAZ AVI) ^e ^	Forest Laboratories (USA)/Astrazeneca	Phase III	Gram-negative	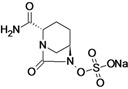 (avibactam)	[142,143,144,145,146,147,148]
	Ceftaroline/Avibactam (CXL)^ e^	Forest Laboratories/ Astrazeneca	Phase II	Gram-negative	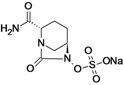 (avibactam)	[120,143,147,148,149,150,151]
	MK-7655 + Imipenem/Cilastatin	Merck & Co., Inc. (USA)	Phase II	Gram-negative	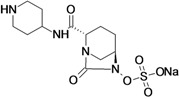 (MK-7655)	[6,147,148,152,153,154,155]
	FPI-1465	Fedora Pharmaceuticals Inc. (USA)	Pre-Clinical	Gram-negative	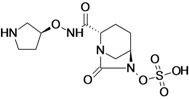	[156,157]
**Beta-lactamase inhibitors—Boronate β-lactamase inhibitor**						
	Carbavance (biapenem + RPX7009)	Rempex Pharmaceuticals (Sweden)	Phase I	Gram-negative	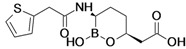 (RPX7009)	[158,159,160,161]
**Beta-lactamase inhibitors—phosphonate-based beta-lactamase inhibitor (BLI)**						
	MG96077	Mirati Therapeutics (USA)	Pre-Clinical	Gram-negative	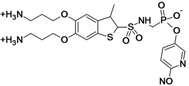	[162,163]
**Lipoglycopeptides**						
	Dalbavancin	Durata Therapeutics (USA)	Phase III completed	Gram-positive	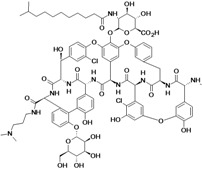	[164,165,166,167,168,169,170,171,172,173]
	Oritavancin Diphosphate	The Medicines Company (USA)	Phase III completed	Gram-positive	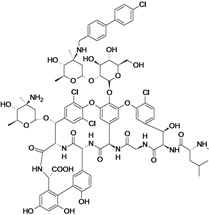	[7,169,170,173,174]
**Polymyxins**						
	NAB739	Northern Antibiotics (Finland)	Pre-Clinical	Gram-negative	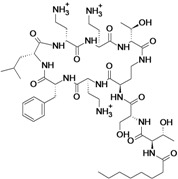	[89,175,176,177,178,179]
	NAB7061	Northern Antibiotics	Pre-Clinical	Gram-negative	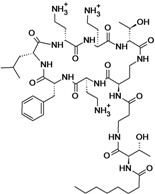	[176,177,178,179]
	NAB741	Northern Antibiotics	Pre-Clinical	Gram-negative	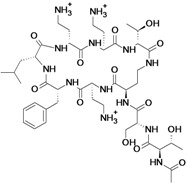	[176,177,178,179]
**Cyclic lipopeptides**						
	Surotomycin	Cubist Pharmaceuticals	Phase III	*C. difficile*	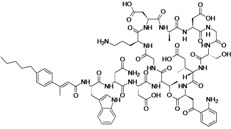	[180,181,182,183]
	WAP-8294A2 (lotilibcin)	Green Cross (South Korea)	Phase I	*S. aureus*	ND	[184,185,186]

ND: Not Divulged; ^a^ The company has not yet published the chemical structure of WCK 2349; ^b^ WCK 771 is the arginine salt of *S*-(−)-nadifloxacin; ^c^ Please find the substituents in the specific claimed compounds on the referenced patents; ^d^ The compound described is the only specific claimed compound in the referenced patent; ^e^ Ceftaroline and ceftazidimeare new cephalosporins already marketed in some countries.

Below, we set forth information about each compound identified and its stage of development.

## 4. Quinolones

There are eleven new quinolones under clinical development and one in the pre-clinical phase of development. The companies pursuing new quinolones are: TaiGen Biotechnology, Kyorin, Ferrer Internacional, Dong Wha Pharmaceutical, Rib-X Pharmaceuticals, Furiex Pharmaceuticals, MerLion Pharmaceuticals, Wockhardt, Daiichi Sankyo, and Achillion Pharmaceuticals. Most of the compounds are fluoroquinolones, but there is one new class of quinolone: isothiazoloquinolones (ITQs).

Nemonoxacin, developed by TaiGen Biotechnology, is a novel nonfluorinated broad-spectrum quinolone. The compound shows greater *in vitro* activity (compared to ciprofloxacin, levofloxacin and moxifloxacin) against Gram-positive bacteria, including resistant pathogens such as methicillin-resistant Staphylococcus aureus (MRSA) and multi-drug-resistant (MDR) *Streptococcus pneumonia*. The activity against most Gram-negative bacteria is similar to that of levofloxacin and moxifloxacin [13].

The company has finished one Phase III clinical study comparing the new compound to levofloxacin for the treatment of community-acquired pneumonia and has submitted a new drug application for the oral formulation of the drug in Taiwan (Taiwan Food and Drug Administration) and China (China Food and Drug Administration) [13,15,17,18]. The compound is also under development for the treatment of diabetic foot infection, for which the company has finished one Phase II clinical trial [16].

Ferrer Internacional has completed one Phase III clinical trial to evaluate the topical formulation of ozenoxacin in the treatment of impetigo [21].

Zabofloxacin is a broad-spectrum antibiotic. The compound shows enhanced activity against both quinolone-susceptible and -resistant *Streptococcus pneumonia* [22,23]. The compound is under development for treatment of chronic obstructive pulmonary disease (COPD) with acute exacerbation. The company is currently recruiting patients for the Phase III clinical trial of oral zabofloxacin compared to moxifloxacin [24,25].

Delafloxacin, developed by Rib-X Pharmaceuticals, is a broad-spectrum fluoroquinolone with improved activity against Gram-positive bacteria when compared to other marketed quinolones. The antibacterial potency of delafloxacin decreases when the pH is slightly reduced, unlike other quinolones such as ciprofloxacin and moxifloxacin, which minimum inhibitory concentration (MIC)’s value increases as the pH decreases. This feature enhances the antibacterial potency in environments with reduced pH such as infection sites that contain inflammatory cells. The compound is active against quinolone susceptible and resistant MRSA strains. The activity against Gram-negative bacteria has shown to be similar to that of ciprofloxacin [26].

The company is currently recruiting patients for a Phase III clinical trial to evaluate delafloxacin compared with vancomycin and aztreonam for the treatment of patients with acute bacterial skin and skin structure infections [26,27,28].

There are three new fluoroquinolones in Phase II of clinical development: avarofloxacin, from Furiex Pharmaceuticals, finafloxacin, from MerLion Pharmaceuticals; and WCK 2349, from Wockhardt.

Avarofloxacin is a novel broad-spectrum fluorinated 4-quinolone. When compared to other marketed fluoroquinolones, the compound shows increased *in vitro* activity against Gram-positive bacteria, including fluoroquinolone-resistant MRSA. The activity of avarofloxacin was equivalent to that of moxifloxacin against Gram-negative bacteria [33,34,35].

In December of 2011, Furiex Pharmaceuticals completed a Phase II study to evaluate avarofloxacin for treating complicated skin and skin structure infections compared to linezolid. The compound is Phase III ready and has received Qualified Infectious Disease Product and Fast Track designations from the US Food and Drug Administration. The company is seeking partners to continue the development of avarofloxacin [29,30,31,32,33,34].

Finafloxacin is a novel fluoroquinolone being developed by MerLion Pharmaceuticals. Under neutral pH conditions (pH 7.2–7.4), the compound has shown *in vitro* activity equivalent to that of ciprofloxacin. However, under slightly acidic pH5.8 the compound shows enhanced potency. Other marketed fluoroquinolones, such as ciprofloxacin, levofloxacin and moxifloxacin, exhibit reduced activity at slightly acidic pH 5.0–6.5. This feature of finafloxacin makes the compound suitable for use in the treatment of infections in acidic foci of infections such as urinary tract infections [39,187,188]. 

MerLion Pharmaceuticals has announced that the FDA has granted a Qualified Infectious Disease Product Designation and Fast Track Status for finafloxacin. The company is currently recruiting patients for the Phase II clinical trial of the compound for the treatment of complicated urinary tract infections (cUTI) and/or acute pyelonephritis compared to ciprofloxacin [37,38]. 

WCK 2349 is a prodrug of WCK 771, which is the hydrate of the arginine salt of *S*-(‒)-nadifloxacin. WCK 771 is a broad-spectrum fluoroquinolone with improved antibacterial activity against quinolone-resistant staphylococci, particularly quinolone resistant MRSA strains [189,190]. 

In June of 2013 Wockhardt completed a Phase II clinical trial in India of WCK 2349 on the treatment of complicated skin and soft tissue infections [40,41,42]. Results have not been published yet.

The other non-fluorinated quinolone under clinical development is KRP-AM1977, by Kyorin, which is in Phase I of clinical trials. The oral formulation of the compound (KRP-AM1977X) is being tested for treatment of respiratory infections and the I.V. formulation is under development for treatment of MRSA infections [44,45]. 

DS-8587, from Daiichi Sankyo, is a fluoroquinolone with improved activity against both Gram-negative and Gram-positive bacteria. The compound is especially effective against *Acinetobacter baumannii* [46,47] but also has improved activity against *streptococci*, *staphylococci*, *enterococci*, *E. coli*, and *anaerobes* [191]. The compound is currently under Phase I of clinical development [47]. 

Achillion Pharmaceuticals is working on the discovery of compounds in a new subclass of quinolones, the isothiazoloquinolones. The most advanced compound is ACH-702, which is at the pre-clinical stage of development [48,192]. 

When compared to levofloxacin, oxacillin and linezolid, the compound shows enhanced *in vitro* activity against Gram-positive bacteria, including fluoroquinolone resistant MRSA. Regarding Gram-negative bacteria, ACH-702 showed improved *in vitro* activity against *Haemophilus influenzae*, *Moraxella catarrhalis*, and *Neisseria* sp. and *anaerobes*. However, it showed less activity against *Enterobacteriaceae* when compared to ciprofloxacin, moxifloxacin, ceftazidime, imipenem and gentamicin [49].

The company is also working on the development of ACH-702 analogs with efficacy against *M. tuberculosis* [48,49].

## 5. Aminoglycosides

There are two companies working on the discovery and development of aminoglycosides: Achaogen and Changzhou Fangyuan Pharmaceutical

Achaogen is pursuing the discovery of new compounds through the optimization of leading compounds from their portfolio of aminoglycosides. The company has published six patent applications for new aminoglycoside compounds in the last two years [53,57].

Also, the company is developing the only aminoglycoside in the clinical stage of development, plazomicin. This compound belongs to a new subclass of aminoglycosides: neoglycosides. It is active against Gram-negative and Gram-positive bacteria and has significantly improved activity against strains resistant to amikacin and/or gentamicin. The compound also shows enhanced activity against resistant *Enterobacteriaceae*, *Acinetobacter*, *Pseudomonas*, and *Staphylococcus*, including Klebsiella pneumoniae carbapenemase (KPC), extended-spectrum beta-lactamase (ESBL) and metallo-beta-lactamase producing *Enterobacteriaceae* and MRSA [51,93,193,194].

A multi-national Phase II study of plazomicin in comparison to levofloxacin for the treatment of complicated urinary tract infections and acute pyelonephritis in adults has been completed. The company announced that that the study met its objectives and the results would be published in an upcoming medical conference [55].

To continue the development of plazomicin, the company has received a contract option of US$ 60M from the Biomedical Advanced Research and Development Authority (BARDA) to support a global Phase III clinical study. The study will evaluate plazomicin in treating patients with serious Gram-negative bacterial infections due to carbapenem-resistant *Enterobacteriaceae*. The study is expected to start in the fourth quarter of 2013 [54].

Changzhou Fangyuan Pharmaceutical is working on the discovery of new aminoglycosides and currently has two compounds in the pre-clinical testing phase: FY-01 to treat *S. aureus* infections, and FY-02 for tuberculosis. The company has published six patent applications for new aminoglycoside compounds in the last two years [57,65].

## 6. Macrolides

Five companies are pursuing the development of new macrolides: Cempra Pharmaceuticals, Enanta Pharmaceuticals, Wockhardt, Basilea Pharmaceutica, and Rib-X Pharmaceuticals.

Cempra Pharmaceuticals is researching new macrolide antibiotics in a library licensed from Optimer. In the last two years the company has published one patent application for new macrolide compounds. The company is developing solithromycin, the first antibiotic from the 2-fluoroketolide subclass. It is the new compound in the most advanced development phase. 

Solithromycin shows improved binding to the ribosomal target and has broad-spectrum activity against macrolide and ketolide-resistant bacteria. The compound is active against the major community-acquired bacterial pathogens, including multi-drug-resistant community-acquired *pneumococci*, *Haemophilus influenzae*, *Moraxella catarrhalis*, and methicillin-susceptible *Staphylococcus aureus* (MSSA) [93]. The compound is active against most off the MRSA strains isolated from community-acquired bacterial pneumonia (CABP), however, MRSA strains with constitutive macrolide resistance are resistant to solithromycin [72].

Macrolides are used in combinations with beta-lactams for the treatment of CABP due to the increasing macrolide resistance in *M. pneumonia*. For the same reason the use of respiratory fluoroquinolones in CABP has increased as a choice of monotherapy [72]. Since it is not possible to treat CAPB with telithromycin not associated with a beta-lactam, the compound is being developed in comparison to moxifloxacin.

The company is recruiting patients for a Phase III clinical study of the oral formulation of solithromycin comparing its activity to that of moxifloxacin for the treatment of patients with community-acquired bacterial pneumonia. It also has one completed Phase II clinical study for the treatment of uncomplicated urogenital gonorrhea with positive outcomes [66,67].

Enanta Pharmaceuticals is working on the discovery and development of bicyclolides (bridged bicyclic macrolides) a new subclass of macrolide antibiotics. The company has published two patent applications for new bicyclolides compounds in the last two years [79,80]. The most advanced compound is EDP-788 (a prodrug of EDP-322) that demonstrates a broad spectrum of activity against many bacterial organisms, including MRSA [75]. 

The compound is currently in pre-clinical development for intravenous and oral formulations, with ongoing investigational new drug (IND) enabling studies. The study is funded under the company’s contract with the National Institute of Allergy and Infectious Diseases (NIAID), with potential for further NIAID funding of early clinical development. The Phase I clinical trials are supposed to begin in 2014 [75,77,78].

Wockhardt is developing a new second-generation ketolide compound, WCK 4873, which is in early pre-clinical development for the oral treatment of infections caused by multi-drug-resistant streptococci, pneumococci and *H. influenzae*. The company has completed regulatory toxicology studies for the compound and is currently in the formulation development phase [81,82].

Basilea Pharmaceutica is pursuing new macrolides using the experience acquired during the identification and characterization of BAL19403, a macrolide compound active against *P. acnes*, with the last stage of development having been reported in 2007 [195].

The RX-02 discovery program at Rib-X Pharmaceuticals is focused on finding new macrolides designed to overcome known ribosomal resistance modifications in a wide range of pathogens. The company is currently seeking partners for the development of this program [83].

## 7. Oxazolidinones

The oxazolidinones are a new class of antibiotics with strong activity against nearly all Gram-positive organisms, including those resistant to other agents. Linezolid was the first member of this class introduced to the market. Although it is an effective antibiotic, new compounds in this class are needed against linezolid-resistant strains and to overcome the side effects caused by the compound.

Seven companies are developing new oxazolidinones: Cubist Pharmaceuticals, Actelion, Rib-X Pharmaceuticals, AstraZeneca, LegoChem Biosciences, MicuRx Pharmaceuticals, Wockhardt.

Cubist Pharmaceuticals is developing tedizolid phosphate (TR-701), a pro-drug of TR-700, which exhibits improved activity against all clinically relevant Gram-positive bacteria, including linezolid resistant *S. aureus* [86]. 

The compound is in a late stage of development, with two completed Phase III clinical studies to evaluate tedizolid compared to linezolid for the treatment of acute bacterial skin and skin structure infections, both trials showing positive results. The company announced that it had submitted a New Drug Application (NDA) to the U.S. Food and Drug Administration (FDA) for approval of the new drug for treatment of acute bacterial skin and skin structure infections (aBSSSi). The company plans to initiate a Phase III study of tedizolid in patients with severe pneumonia during the second half of 2013 [88,196,197,198].

Cadazolid is a new oxazolidinone with a fluoroquinolone side chain (hybrid antibiotic) [89,91]. The compound has shown to have potent activity against *Clostridium difficile* bacteria and is being developed by Actelion for treatment of *Clostridium difficile*-associated diarrhea (CDAD). The company completed a Phase II clinical study in 2012 to an oral formulation of Cadazolid for treatment of CDAD and is planning to start a Phase III study in the fourth quarter of 2013 [90,92].

Rib-X Pharmaceuticals has completed two Phase II clinical trials of radezolid for the treatment of pneumonia and uncomplicated skin infections. The trial completion dates were in 2008 and 2009, but to date the Phase III trials have not been initiated [93,94,95,96,97].

AstraZeneca is currently pursuing the development of AZD5847, a new oxazolidinone active against Gram-positive bacteria and mycobacteria. AZD5847 is active against a broad array of drug-sensitive and drug-resistant strains of *Mycobacterium tuberculosis*. The compound is under Phase IIa clinical development to assess early bacterial activity of the compound. The company is currently recruiting participants to be part of the trial [99,100,101].

LCB01-0371 is being developed by LegoChem Bio. This new oxazolidinone has improved activity against Gram-positive pathogens and has good pharmacokinetic profiles in animals [103]. 

The compound is under Phase I clinical development to assess the safety and tolerability of the compound. The company is currently recruiting participants to be part of the trial [103,104].

MicuRx Pharmaceuticals is developing two oxazolidinone compounds MRX-I and MRX-II. MRX-I is an oral oxazolidinone antibiotic that targets infections due to resistant Gram-positive bacteria, including MRSA and vancomycin-resistant enterococci (VRE). The company announced the completion of a double-blinded, placebo-controlled Phase 1 clinical study, and that the compound has been shown to be safe and well-tolerated at all doses tested with no evidence of myelosuppression. In October 2012, the company announced the establishment of Shanghai MengKe Pharmaceuticals, a joint venture with Shanghai Zhangjiang Biomedical Industry Venture Capital formed to fund the development and commercialization of MRX-I for the Chinese market. MRX-II is currently under pre-clinical development [105,106].

Wockhardt is also working on the development of second-generation oxazolidinones, which are in pre-clinical testing for Gram-positive infections [81].

## 8. Tetracyclines

Two companies were found to have tetracycline antibiotic R&D: Paratek and Tetraphase.

Paratek is developing a new subclass of tetracyclines called aminomethylcyclines. Omadacycline, the lead compound, is a broad-spectrum antibiotic with improved activity against tetracycline-resistant pathogens, including methicillin-susceptible and -resistant *staphylococci* as well as *streptococci* and *enterococci*. The compound is also active against many clinically important *Enterobacteriaceae* and a wide range of anaerobes [199].

The compound is currently under development for the treatment of ABSSI, CABP and UTI. The company had started a Phase III trial for complicated skin and skin structure infections (cSSSI) in March 2010, when the FDA notified that it would modify the guidance on the conduct of studies for this indication. With these modifications, the Phase III trial design did not align with the FDA’s then-evolving guidance for trials aimed at supporting the approval of an antibiotic for the treatment of aBSSSI. As a result, the Phase III trial was terminated after having enrolled 140 of the planned 790 subjects. 

The FDA has since granted Omadacycline Qualified Infectious Disease Product (QIDP) designation for oral and IV formulations in the treatment of aBSSSi and CABP. The Phase III studies will be conducted under two special protocol assessment agreements, one for aBSSSI and one for CABP. Paratek is currently planning additional studies of omadacycline for the treatment of urinary tract infection. The company plans to simultaneously conduct two Phase III registration trials for aBSSSI, and submit an NDA for the treatment of aBSSSI in late 2014 [200]. 

Tetraphase Pharmaceuticals is developing a new class of synthetic tetracyclines, called fluorocyclines. The company has published three patent applications for new tetracycline compounds in the last two years [116,117,118]. The lead compound is eravacycline, a broad-spectrum fluorocycline antibiotic with activity against bacteria expressing major antibiotic resistance mechanisms, including tetracycline-specific efflux and ribosomal protection. The compound has improved activity against many MDR Gram-negative bacteria, including beta-lactam resistant *E. coli*, *K. pneumoniae* and *Acinetobacter* spp*.*

The company successfully completed a Phase II clinical study in 2012 to evaluate the safety of the intravenous formulation of eravacycline for the treatment of cIAI, and started a Phase III study in August of 2013 [201]. They announced that they expect to begin the cUTI Phase III clinical trial in the fourth quarter of 2013 [202].

The company has two other fluorocyclines in the pipeline, both in pre-clinical testing: TP-271, for the treatment of bacterial biothreats and CABP pathogens, and TP-834, active against CABP pathogens. The company is also working to discover novel tetracycline derivatives or treatment of Gram-negative infections [109,113,114].

## 9. Pleuromutilins

Pleuromutilins have been known since 1951, but only entered the market in 2007 with the approval of retapamulin for topical use. Until today, there are no pleuromutilins for systemic use approved in human clinical practice.

Nabriva is currently working on the development of new compounds is this class. The lead compound, BC-3781, if approved, will be the first pleuromutilin for systemic use in humans. 

The compound shows potent *in vitro* activity against a large collection of *staphylococci*, *streptococci*, and *E. faecium.* When compared to linezolid and vancomycin, the compound shows greater overall potency against *S. aureus* [121]. BC-3781 shows improved activity against most bacteria commonly associated with community-acquired respiratory tract infections, the compound is especially potent against *S. pneumonia* including penicillin resistant strains. It also shows improved activity against *H. influenza*, *M. catarrhalis*, *M. pneumoniae* and *C. pneumoniae*.

BC-3781 is undergoing Phase I clinical trials for CAP and in March of 2011 has completed a Phase II clinical study comparing it to vancomycin for treatment of aBSSSI [119,120,121,122,123]. Nabriva Therapeutics AG announced that the cooperation with Forest Laboratories to develop the compound had elapsed, and that Nabriva retained all rights in BC-3781. The company informed that the product was Phase III ready and that it was seeking partners to continue further development [203].

Nabriva is also developing BC-7013 for topical use against Gram-positive infections and working on the discovery of new pleuromutilins [119,124].

## 10. Beta-Lactams

BAL30072 is a novel monosulfactam under clinical development. The compound has shown improved *in vitro* activity against significant Gram-negative pathogens especially against *A. baumannii* where it has shown to be active against meropenem-resistant strains. The compound also presents improved activity against *P. aeruginosa*, *Burkholderia cepacia*, and some MDR *Enterobacteriaceae*, however, it shows lower potency against ESBL-producing Enterobacteriaceae [4,204].

Basilea Pharmaceutica is conducting Phase I studies to assess the pharmacokinetics, safety and tolerability of BAL30072. The company announced that it had received a contract of USD17 million with BARDA, to continue the Phase 1 studies of safety and tolerability of BAL30072 alone and in combination with carbapenems [133,134,135].

There are four cephalosporins under development. The cephalosporin in the most advanced phase of development is ceftolozane a novel oxyimino-aminothiazolyl cephalosporin, which is under development in combination with tazobactam to treat infections by Gram-negative pathogens. 

The ceftolozane/tazobactam combination has demonstrated improved *in vitro* activity against *P. aeruginosa* (including to MDR strains) when compared to ceftazidime and cefepime. The combination is also more active than ceftazidime and piperacillin-tazobactan against ceftazidime-resistant *Enterobacteriaceae* species. However, ceftolozane/tazobactam is less active than cefepime against some species of ceftazidime-resistant Enterobacteriaceae [205]. 

The company is recruiting patients for four ongoing Phase III clinical trials: two comparing the ceftolozan/tazobactam combination with levofloxacin for treatment of cUTI and another two comparing the combination with meropenem for treatment of cIAI [6,128,129,130,131].

GlaxoSmithKline is developing S-649266 (GSK2696266) with Shionogi. The compound is active against Gram-negative bacteria and is has strong anti-bacterial activity against New Delhi metallo-beta-lactamase-1 (NDM-1) producing bacteria, which have developed resistance to many carbapenem and cephem antibiotics [137]. S-649266 is currently in Phase I of clinical trials [136,137,138].

The cephalosporin under pre-clinical development is CB-027, being developed by Cubist Pharmaceutical, which has shown potent *in vitro* activity against both Gram-positive and Gram-negative bacteria including MRSA and *Pseudomonas aeruginosa*. The *in vivo* activity against clinical isolates of MRSA was shown to be comparable to vancomycin and ceftaroline. CB-027 also had good activities against ceftazidime-resistant *Pseudomonas aeruginosa* and *Klebsiella pneumonia* [139,206].

FOB Synthesis, Inc. is developing FSI-1671, a novel carbapenem. The compound shows improved *in vitro* activity against *A. baumannii*, including to MDR-strains, and the FSI-1671/sulbactam combination showed synergy against MDR-*A. baumannii* [140,141]. FOB synthesis presented the results of the pre-clinical studies in the 53rd International Interscience Conference on Antimicrobial Agents and Chemotherapy, but has not informed if will pursue further development of the compound.

## 11. Beta-Lactamase Inhibitors—Diazabicyclooctanes (DBOs)

Diazabicyclooctanes (DBOs) are a rich source of β-lactamase inhibitors (BLI). Two members of this novel series are now in clinical development, avibactam and MK-7655. Both compounds are broad-spectrum beta-lactamase inhibitors and are more potent compared to tazobactam [147,148].

Avibactam is being developed by Forest Laboratories and Astrazeneca in combination with ceftazidime and ceftaroline for the treatment of Gram-negative infections. The compound demonstrated *in vitro* activity against Ambler class A and C β-lactamases, including KPC, and some class D enzymes [207].

The use of avibactam with ceftazidime improves the activity of the compound against most species of beta-lactamase producing *Enterobacteriaceae* and against *Pseudomonas aeruginosa* [208].

This feature makes the combination ceftazidime/avibactam suitable for treatment of infections with resistant Gram-negative bacilli-producing ESBL, KPC and/or AmpC beta-lactamases and for treatment of *P. aeruginosa* infections [208].

Five Phase III clinical trials are currently recruiting patients to evaluate the use of ceftazidime/ avibactam for the treatment of complicated urinary tract infection, complicated intra-abdominal infection, nosocomial pneumonia and ventilator-associated pneumonia [142,143,144,145,146,147,148].

The combination ceftaroline/avibactam has shown activity equivalent to that of ceftazidime/avibactam with two exceptions, the activity against MRSA and limited activity against *P. aeruginosa* [209].

In July 2012, the companies completed the Phase II clinical study of intravenous ceftaroline fosamil/avibactam compared to intravenous doripenem in adults with complicated urinary tract infection. Phase III has not yet started [120,143,149,150,151].

Merck & Co., Inc. is currently developing MK-7655, to be used in combination with imipenem/ cilastatin. MK-7655 demonstrated potent *in vitro* inhibition of class A and class C β-lactamases [155]. 

The compound improves the activity of imipenem against *Enterobacteriaceae* producing KPC carbapenemases or combinations of β-lactamase and impermeability. It does not improve the activity of imipenem against metallo-carbapenemases producing *Enterobacteriaceae*. The activity of imipenem against *P. aeruginosa* generaly improved, particularly OprD mutants [210].

There are two Phase II clinical studies currently recruiting patients to evaluate the combination of MK-7655 + imipenem/cilastatin compared to imipenem/cilastatin alone in the treatment of cUTI and cIAI [152,153,154,155].

Fedora Pharmaceuticals Inc. is developing FPI-1465, which is currently under pre-clinical testing. Preliminary *in vitro* tests have shown that the compound inhibits ESBL enzymes of several *Enterobacteriaceae* species such as CTX-M and plasmid AmpC. The compound has demonstrated synergistic effects with aztreonam and ceftazidime [156,211]. The company has announced that it expects to file an Investigational New Drug (IND) application and initiate clinical studies in 2014 [157].

## 12. Beta-Lactamase Inhibitors—Boronate β-Lactamase Inhibitor

Rempex Pharmaceuticals is developing carbavance, a combination of biapenem and a new beta-lactamase inhibitor, RPX7009, for the treatment of Gram-negative infections. The combination is in Phase I of clinical trials [158,159,160].

## 13. Beta-Lactamase Inhibitors—Non-beta-Lactam Phosphonate-Based

Mirati Therapeutics is seeking partners to continue the development of the compound MG96077, a non-beta-lactam phosphonate-based beta-lactamase inhibitor that has shown an inhibitory profile for both class A and class C beta-lactamase enzymes [162,163].

## 14. Glycopeptides (Lipoglycopeptides)

Telavancin is the first approved compound of the lipoglycopeptides, a new class of antibiotics. Two other lipoglycopeptides are in Phase III of clinical development: dalbavancin, by Durata Therapeutics, and oritavancin diphosphate, by The Medicines Company. Both are in development for the treatment of aBSSSi. 

All three compounds demonstrate bactericidal activity against MSSA, MRSA, methicillin-sensitive *Staphylococcus epidermidis* (MSSE), methicillin-resistent *Staphylococcus epidermidis* (MRSE) and penicillin-resistant *S. pneumonia*, therefore may be used for the treatment of resistant Gram-positive pathogens replacing vancomycin in cases of resistance or intolerace of vancomycin adverse effects [212].

Durata Therapeutics has completed two global Phase III clinical trials of dalbavancin for the treatment of acute bacterial skin and skin structure infections caused by Gram-positive bacteria, and is preparing a new drug application to the FDA [164,168]. 

The Medicines Company completed the second Phase III clinical trial of oritavancin diphosphate for the treatment of aBSSSi in December 2012 [164,165,166,167,168,169,170,171,172,173]. The company announced that it plans to submit a New Drug Application to the FDA in the fourth quarter of 2013 [170,171,213].

## 15. Polymyxins

Polymyxins act by interacting with the phospholipids of the cell membrane and disrupting it, causing permeability leading to bacterial death. These compounds are active only against Gram-negative bacteria. Their systemic use is not recommended because of toxicity reasons, but there has been a resurgence in the systemic use of polymyxins due to the emergence of multidrug-resistant Gram-negative organisms [214]. 

The only company identified that has polymyxins in the pipeline is Northern Antibiotics, which is conducting pre-clinical testing of three polymyxin compounds. NAB739 has shown improved antibacterial activity against Gram-negative pathogens. The other two compounds, NAB7061 and NAB741, lack direct activity but improve the activity of other antibiotics against these bacteria when used together [175,176,177]. 

NAB739 has demonstrated activity similar to that of polymyxin B against a set of clinical isolates of *E. coli*. However it has shown an overall narrower antibacterial *in vitro* profile than polymyxin B, because of the decreased activity against *Acinetobacter* spp. and weaker activity against *P. aeruginosa.* The clinical importance of the NAB739 compound will be determined by studies comparing the nephrotoxicity of NAB739 and polymyxin B *in vivo* and the *in vivo* activity of the compound against Gram-negative bacteria [175].

## 16. Cyclic Lipopeptides

Cyclic lipopeptides, with activity against Gram-positive bacteria, are a new class of antibiotics in clinical practice, and daptomycin is the first and only antibiotic in this class. This compound works by binding to bacterial membranes, which results in depolarization, loss of membrane potential, and cell death [214]. 

Two cyclic lipopeptides were identified in the clinical pipeline: surotomycin, from Cubist Pharmaceuticals, and WAP-8294A2 (lotilibcin), from Green Cross.

Surotomycin, developed by Cubist Pharmaceuticals, is structurally related to daptomycin and shares the mechanism of action against *Clostridium difficile* [183].

The company is currently recruiting patients for a Phase III clinical study comparing the new compound to vancomycin in the treatment of CDAD [180,181,182,183].

Green Cross is conducting a Phase I clinical trial to assess the safety of the new compound WAP-8294A2 (lotilibcin), under development for the treatment of infections caused by *S. aureus* [184,185,186].

## 17. Final Remarks

This article maps out the R&D of new antibiotics from known chemical classes, from the discovery stage to clinical trials. Many compounds were identified at the pre-clinical and development stages, but there is very little information divulged at such early stages of development.

Concerning the compounds under clinical testing, of the ten antibiotic classes identified, seven had compounds at Phase III, namely: quinolones (nemonoxacin for CAP; delafloxacin for aBSSSi), macrolides (solithromycin for CAP), oxazolidinones (tedizolid for aBSSSi; omadacycline for CAP, aBSSSi and UTI), cephalosporins (ceftolozane + tazobactam for cUTI and cIAI), lipoglycopeptides (dalbavancin for aBSSSi; oritavancin diphosphate for aBSSSi) and cyclic lipopeptides (surotomycin for CDAD). 

Quinolones are the leading antibiotic class, with nine compounds under clinical testing by six companies from different parts of the world (Taiwan, Japan, South Korea, USA, India). Oxazolidinones are also an important class, with three compounds in clinical trials and five companies working in R&D.

Interestingly, of the chemical classes identified, only oxazolidinones, lipoglycopeptides and cyclic lipopeptides are recent classes (new compounds since 2000); the others are classes discovered prior to the 1970s.

Concerning the activity of the compounds, it can be seen that the recent trend in new antibiotic development has been on the development of new compounds in known chemical classes for the treatment of Gram-positive bacterial infections, which is reflected in the great number of compounds for aBSSSI and CABP undergoing clinical trials.

Among the compounds in clinical testing, only a few showed enhanced activity against MDR-Gram-negative bacteria. Plazomicin and eravacycline have shown a broad-spectrum activity against most MDR-Gram-negative bacteria. However, most of the development against Gram-negative bacteria is relying on the development of new and more potent beta-lactamase inhibitors to partly restore the activity of known antibiotics. It is uncertain if this approach will be effective to overcome serious MDR-Gram-negative infections. If this approach fails, there are only a few other new compounds from known classes under development to treat these types of infection.

The eight compounds in Phase III mapped out in the present article show that it is likely that in the near future the new chemical compounds from known classes will be very important for treating infections by increasingly resistant bacteria. 

It is concluded that many companies are still keen to develop new antibiotics by modifying compounds from existing chemical classes. The number of companies pursuing such an approach in their R&D at different stages indicates that this kind of research is very important and will be a reality for a good while yet.
